# Peptide Regulation of Chondrogenic Stem Cell Differentiation

**DOI:** 10.3390/ijms24098415

**Published:** 2023-05-08

**Authors:** Natalia Linkova, Vladimir Khavinson, Anastasiia Diatlova, Svetlana Myakisheva, Galina Ryzhak

**Affiliations:** 1 Saint Petersburg Institute of Bioregulation and Gerontology, Dynamo pr. 3, 197110 Saint Petersburg, Russia; 2 Pavlov Institute of Physiology of Russia Academy of Sciences, Makarova emb. 6, 199034 Saint Petersburg, Russia

**Keywords:** osteoarthritis, mesenchymal stem cells, chondrogenic differentiation, peptides, growth factors

## Abstract

The search for innovative ways to treat osteoarthritis (OA) is an urgent task for molecular medicine and biogerontology. OA leads to disability in persons of middle and older age, while safe and effective methods of treating OA have not yet been discovered. The directed differentiation of mesenchymal stem cells (MSCs) into chondrocytes is considered one of the possible methods to treat OA. This review describes the main molecules involved in the chondrogenic differentiation of MSCs. The peptides synthesized on the basis of growth factors’ structures (SK2.1, BMP, B2A, and SSPEPS) and components of the extracellular matrix of cartilage tissue (LPP, CFOGER, CMP, RDG, and N-cadherin mimetic peptide) offer the greatest promise for the regulation of the chondrogenic differentiation of MSCs. These peptides regulate the WNT, ERK-p38, and Smad 1/5/8 signaling pathways, gene expression, and the synthesis of chondrogenic differentiation proteins such as COL2, SOX9, ACAN, etc.

## 1. Introduction

Osteoarthritis (osteoarthritis deformans and osteoarthrosis; ICD-10: M15-M19 Arthrosis, with the exception of M47 (osteoarthritis of the spine)) is a widespread disease characterized by damage to the arthrodial cartilage adjacent to the bone and soft tissues of a joint. Osteoarthritis is characterized by progressive irreversible structural changes in the cartilage and a pronounced pain syndrome. In the Russian-language literature, the term “osteoarthrosis” is used, while in the foreign-language literature, “osteoarthritis” is a more common term to refer to the same disease, since, in most cases, the disease is accompanied by signs of inflammation. Osteoarthritis (OA) mostly affects people of middle and older age. The disease is predominantly diagnosed at the age of 40–50 years. Early diagnosis of osteoarthritis (in patients under 40 years of age) is rare since the initial stage of the disease is asymptomatic. The incidence of OA is higher among women aged 40–70 years than among men of the same age group. After 70 years of age, the incidence of OA is the same for both genders. The clinical pattern of OA includes deep, dull pain that worsens with physical activity and subsides at rest. As the disease progresses, the pain does not subside at rest and continues even at night, which may lead to partial or complete disability. The main causes of OA development include microtraumatization of cartilaginous tissue, a decrease in proteoglycan aggregation during aging, various genetic factors, chronic inflammation, obesity, neuropathies, and accumulation diseases (a heterogeneous group of hereditary diseases with a severe course including, in particular, sphingolipidoses (Gaucher’s disease and Fabry disease), mucopolysaccharidoses (Hunter’s syndrome), oligosaccharidoses, mucolipidoses, lipidoses, etc.) [1].

The existing methods for OA treatment include pain relief with non-steroidal anti-inflammatory drugs (NSAIDs) and rehabilitation exercises. In acute pain and complications, intra-articular corticosteroids are used; the effects of hydroxychloroquine administration have been tested on small samples [2]. Due to the prevalence of OA among middle-aged and older people, it is necessary to develop new therapeutic methods aimed at maintaining the structure and normalizing the functional activity of cartilage tissue.

Methods for the regeneration of cartilage tissue are very promising for the treatment of OA. The following methods have been studied experimentally in order to investigate their feasibility for the treatment of focal lesions in the articular cartilage of the knee joint: the implantation of autologous chondrocytes (ACI) and its modification, MACI, mosaic chondroplasty; the chondrogenic differentiation of mesenchymal stem cells; administration of platelet-rich plasma, growth factors, and bone morphogenetic proteins (BMPs); the application of elastic-like polypeptide gels; titanium implants coated with stem cells; and chondroprotection with pulsed electromagnetic fields [3]. The above methods demonstrate different efficacies and require further study.

Mesenchymal stem cells (MSCs) represent a promising resource for cellular biotechnology. Protocols have been developed to obtain MSCs from peripheral blood, bone marrow, adipose tissue, etc. [4,5]. Classically, MSCs differentiate into osteoblasts, chondroblasts, adipocytes, and fibroblasts, but their ability to differentiate into other cell types has also been observed. The addition of special factors to a culture medium during MSC cultivation directs their development along the chondrogenic differentiation pathway. In 2003, the first joint surgeries using bone marrow MSCs (BM-MSCs) were performed. The pre-transplant cultivation of MSCs aimed at the accumulation of cell biomass is one of the necessary and key stages of restorative cell therapy. However, it has not yet been possible to grow large amounts of MSCs with a fully preserved differentiation potential under in vitro conditions. Therefore, the study of the molecular cascades of the chondrogenic differentiation of stem cells remains relevant for the development of new methods for cartilage tissue repair and the modification of existing ones.

Short peptides have biological activity that has been demonstrated in a wide range of experiments [6]. The properties of short peptides include the regulation of proliferation [7], differentiation [8,9], apoptosis [10,11,12], innate immunity [13,14,15], tumorigenesis [16,17], and wound healing [18,19,20]. Results on the regulation of chondrogenic differentiation via various types of short peptides have been published [21,22,23], so it should be relevant to discuss these bioactive molecules in the context of the chondrogenic differentiation of MSCs.

In this regard, the aim of this review is to analyze the molecular aspects of the chondrogenic differentiation of stem cells as targets for cartilage tissue repair in OA, as well as to analyze the peptides that can regulate this process.

## 2. Materials and Methods

The PubMed database was used to search the relevant references. The keywords included “mesenchymal stem cells”, “chondrogenic differentiation”, and “short peptides in chondrogenic differentiation”. The search was performed from December 2022 to March 2023.

## 3. Differentiation of Human Chondrocytes

The source of cartilage tissue in embryonic development is the skeleton-forming sclerotome mesenchyme, which gives rise to a population of pluripotent skeletal mesenchymal cells (PSCMs). PCSMs give rise to bipotent osteochondroprogenitor cells with osteogenic and chondrogenic potentials. One of the pathways of PCSM differentiation is via osteoblasts and the further formation of flat bones, for example, skull bones. Another pathway of bone formation is the endochondral way, that is, through intermediate cartilage. In this case, osteochondroprogenitor cells form a precartilaginous mass, the inner cells of which differentiate into early chondrocytes, while the cells at the periphery of the mass remain unused. Early chondrocytes undergo stages of differentiation, flatten, and fold into longitudinal columns to form growth plates. Early chondrocytes actively proliferate until they reach the pre-hypertrophic stage, wherein the osteogenesis of cells located on the periphery of the precartilaginous mass (perichondrium) occurs. The pre-hypertrophic stage is followed by hypertrophy and the apoptosis of chondrocytes. Osteoblasts, endothelial cells, osteoclasts, and hematopoietic cells migrate from the perichondrium to the area of chondrocyte localization, forming bone and bone marrow tissues instead of cartilage [24].

In addition to osteochondroprogenitor cells, skeleton-forming mesenchymal progenitors can differentiate into articular chondrocytes (articular cartilage chondrocytes) through the stage of multipotent articular progenitors, giving rise to synoviocytes [24].

Each stage of osteoblast and chondrocyte differentiation is characterized by the expression of certain genes: early chondrocytes express *Col2a1* (type II collagen) and *Acan* (aggrecan); columnar cells express *Fgfr3*; pre-hypertrophic cells express *Ppr* (parathyroid hormone-related protein receptor), *Ihh* (Indian hedgehog), and *Col10a1* (type X collagen); and hypertrophied cells express *Col10a1*. Terminal chondrocytes mineralize the extracellular matrix, with *Mmp13* (matrix metalloproteinase 13) and *Bsp* (bone sialoprotein) serving as their markers [25].

It has been established that in vitro MSCs can form a cartilage-like tissue containing biomolecules that are typical of cartilage: type II collagen, proteoglycans, and aggrecan [26]. The most studied are BM-MSCs. The study of MSCs from the postpartum placenta (P-MSCs) seems promising due to the availability of biomaterial and high proliferative potential. Zhernosechenka et al. (2021) conducted a comparative analysis of the chondrogenic and osteogenic potential of BM-MSCs and P-MSCs. For their chondrogenic differentiation, the authors cultivated cells in pellets (a 3D system) in Dulbecco’s Modified Eagle Medium (DMEM) containing TGFβ and IGF1. After directed induction, the main chondrogenic markers *Runx2*, *Sp7*, *DMP*, *Coll2*, *Coll1*, *Coll10*, *COMP*, and *Ver* were analyzed via the polymerase chain reaction (PCR) method. It was shown that among the genes responsible for collagen synthesis, the expression of *Coll2* was 18 times higher, and *Coll10* was 4 times higher in predifferentiated BM-MSCs than in P-MSCs. Thus, more intensive synthesis of type II and X collagen was observed in BM-MSCs. The expression of the *COMP* gene, responsible for the synthesis of the oligomeric cartilage matrix protein that binds collagen fibers, was more than six times higher in predifferentiated P-MSCs compared with BM-MSCs. The expression of the versican gene from the aggrecan family, *Ver*, was two times higher in predifferentiated P-MSCs compared with BM-MSCs. The content of mucopolysaccharides was also higher in the P-MSC culture than in the BM-MSC cultures [27].

Bernardo et al. (2007) obtained similar results regarding the contents of different types of collagens after MSC differentiation by means of immunohistochemistry. After chondrogenic differentiation, the synthesis of type II, IX, and X collagens in human BM-MSCs increased [28].

However, the tissue-engineered cartilage did not achieve the structure and functionality of the native cartilage. The collagen content in the artificial cartilage was 50% lower than in the native one. The ultrastructure and spatial organization of the artificial cartilage fibers also differed from the native ones, which affected their mechanical properties [29]. The disadvantage of artificial cartilage is the acquisition by differentiated chondrocytes of a hypertrophied phenotype, characterized by a significant increase in metabolic activity, the deposition of large amounts of extracellular matrix (ECM), including type X collagen, and the production of alkaline phosphatase (an enzyme involved in matrix mineralization), and their subsequent apoptosis, which leads to a decrease in its functional activity [30]. Current studies are aimed at improving the understanding of chondrogenic differentiation processes and optimizing the available MSC cultivating strategies for cartilage tissue engineering.

## 4. MSC Chondrogenic Factors

In vitro chondrogenic differentiation requires two conditions: the presence of growth factors (bFGF, TGF-β3, and BMP-2) in the culture medium and a 3D culture system. In vitro, chondrogenesis is estimated according to the gene expression of aggrecan, transcription factor Sox9, and type II collagen (*Col2a1*) using quantitative PCR or immunohistochemistry. Chondrogenic differentiation is accompanied by the appearance of HLA-DR molecules on the membranes of chondrocyte precursors [31].

TGF-β1, 2, and 3 are the only established factors of chondrogenesis, the addition of which to a culture medium leads to the accumulation of proteoglycan and type II collagen [32]. It is assumed that the dysregulation of TGFβ signaling in MSCs may be one of the causes underlying OA development. It has been shown that TGF-β1 ensures the proliferation of antler chondrocytes and induces the expression of hypertrophied chondrocyte markers, such as type X collagen, Runx2, and Alpl, via the Notch-Shh-Foxa signaling pathway. A wide range of methods, including immunofluorescence, Western blot, MTS, flow cytometry, RNA interference, and PCR-RT were used in that investigation [33]. TGF-β activates chondrogenic differentiation partly through a mediated decrease in N-cadherin synthesis, which leads to a decrease in the activation of the RhoA/ROCK signaling pathway, which has been shown in the BMSC line using qPCR [34].

Transcription factors (TFs) control the differentiation of chondrocytes at the level of gene expression. Some chondrogenic transcription factors, such as SOX9 and RUNX2/3, play a major role in certain stages of differentiation, while others, such as SOX5/6 and MEF2C/D, can maintain or decrease the activity of the main transcription factors. The regulators of chondrogenic differentiation include growth factors that regulate the proliferation and differentiation rate [24].

TFs with β-scaffolds include SOX proteins that bind DNA along the C[A/T]TTG[A/T][A/T] motif. The *SOX9* gene is expressed during chondrogenesis starting from the stem cell stage. It regulates differentiation at the stage of prechondrocytes, early chondrocytes, growth plate chondrocytes, and articular cartilage chondrocytes, that is, embracing almost all stages of differentiation. It is assumed that in columnar chondrocytes, *SOX9* suppresses the expression of *Col10a1*, and in prehypertrophied and hypertrophied chondrocytes, *SOX9* is essential for the expression of *Col10a1*. In addition, *SOX9* supports aggrecan synthesis in mature articular cartilage [24].

The *Sox5* and *Sox6* genes are co-expressed in chondrocytes with *Sox9*. The structure and functions of the SOX5 and SOX6 proteins are similar since they belong to the same SOXD family. However, they have only 50% identity with SOX9, which belongs to the SOXE family. Knockout of the *Sox5* gene or *Sox6* gene leads to moderate skeletal disorders in neonatal mice. Knockout of both of these genes leads to intrauterine fetal death due to severe chondrodysplasia [35].

Knockout of the *Sox8* gene leads to osteopenia in mice; however, malformations of cartilage tissue have not been observed [36]. SOXC proteins (SOX4, SOX11, and SOX12) are important for maintaining the life cycle of MSCs. They induce WNT-dependent differentiation along the osteoblast line and are involved in chondrogenic differentiation as supporting factors shown in embryogenesis in mice [37].

In the absence of WNT pathway signals, proteins of the TCF/LEF (T-cell factor/lymphoid enhancer factors) family, including TCF7 (TCF1), TCF7L1 (TCF3), TCF7L2 (TCF4), and LEF1, suppress gene expression. In classical WNT signaling, the transcription coactivator β-catenin translocates to the nucleus, binds to TCF/LEF factors, and converts them into transcription activators [38].

Expression of the TCF7L2 protein has been demonstrated in hypertrophied chondrocytes. These chondrocytes interact with RUNX2, which has been shown using RT-PCR and an immunoprecipitation assay [39]. Apart from that, the TCF7L2 protein is expressed in chondrocyte cell cultures obtained from humans with primary OA and enhances the apoptotic effects of NFκB [40].

Proteins of the RUNX family (also called CBFs, core-binding factors) consist of α and β subunits encoded by the RUNX1/CBFA2, RUNX2/CBFA1, RUNX3/CBFA3, and CBFB genes. The affinity of the α subunit, which binds to DNA along the [A/G]ACC[A/G]CA motif, is higher than that of the β subunit. The RUNX2 and RUNX3 proteins are the key regulators of chondrogenesis. Mice with a knockout *Runx2* gene die at birth; they do not have intramembrane and endochondral ossification and lack prehypertrophied and hypertrophied chondrocytes. Interactions between RUNX2 and RUNX3 may be important for the formation of mature chondrocytes. The absence of *Runx3* expression did not affect the formation of the bone growth plate. However, complete knockout of the *Runx2* and *Runx3* genes resulted in the absence of maturing chondrocytes in mice. RUNX2 and RUNX3 are transcription activators targeted at markers of prehypertrophied (*Ihh*), hypertrophied (*Col10a1*), and terminal (*Mmp13*) chondrocytes [41]. *Runx1* expression was observed in the articular cartilage tissue of adults with OA [42].

MEF2 proteins are transcription factors with MADS (MCM1, AGAMOUS, DEFICIENS, and SRF, serum response factor) domains. They are transcription activators involved in muscle development and are involved in other processes, including chondrogenesis [43]. The MEF2C and MEF2D proteins have been shown to co-express in the growth plate during mouse embryogenesis. In combination with RUNX2 and SOX9, they enhanced *Ihh* and *Col10a1* expression in prehypertrophied and hypertrophied chondrocytes in mice, which was demonstrated using an electrophoretic mobility shift assay and chromatin immunoprecipitation [44,45].

The NFkB and NFATC protein families belong to TFs with RHD domains and include several chondrogenic proteins. Members of the NFkB family bind DNA along the GGG[A/G]N[T/C][T/C][T/C]CC motif. The activation of NFkB proteins is induced by stress and inflammatory signals, as a result of which *Sox9* expression is suppressed and catabolic processes in chondrocytes are triggered. On the contrary, one of the proteins of the RELA/p65 family is expressed in healthy MSCs and growth plates and promotes cell survival and proliferation in vitro [46].

Seven proteins of the STAT family regulate cell proliferation, differentiation, and survival. Modulated via the fibroblast growth factor receptor, FGFR3, STAT1, and STAT5 inhibit the proliferation of columnar chondrocytes, which has been demonstrated in achondroplasia and thanatophoric dysplasia tissue, as well in chondrocyte cultures obtained from humans suffering from these diseases [47]. STAT5 mediates the action of growth hormones on the growth plate. The in vivo stimulation of STAT3 via pro- or anti-inflammatory cytokines leads to changes in chondrocyte differentiation [46].

Other molecular factors that induce the chondrogenic differentiation of MSCs include activin, osteogenic protein-1, GDF-5, IGF-1, prolactin, interleukin (IL)-1β, Cyr61, HB-GAM, somatotropin, and concavalin A [48].

**Figure 1:**
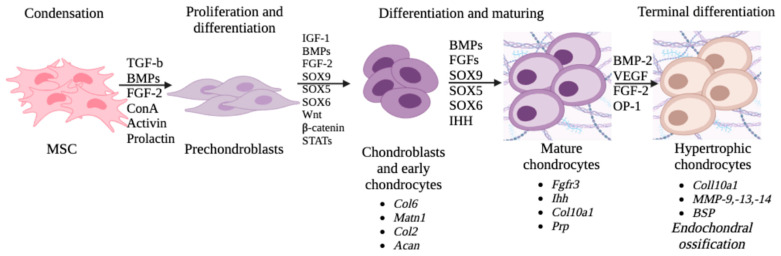
Chondrogenic differentiation of MSCs: signaling pathways and molecular markers.

Thus, chondrogenesis is due to the condensation and differentiation of MSCs in several stages, each of which is controlled by TFs. Chondroblasts, differentiating into early and then into mature chondrocytes, synthesize components of the extracellular matrix that are characteristic of cartilage tissue, in particular, type II and type VI collagen (*Col2* and *Col6*), aggrecan (*Acan*), and matriline 1 (*Matn1*). The markers of mature chondrocytes are *Ihh*, *Fgfr3*, and *Coll10a1*. The hypertrophic zone is characterized by the expression of type X collagen, calcification, and matrix remodeling with the participation of matrix metalloproteases MMP-9, -13, and -14. Vascularization mediated via vascular endothelial growth factor (VEGF) and VEGF receptors is required for the conversion of unvascularized tissue into bone. All these processes are regulated via FGF, TGF, bone morphogenetic protein (BMP), the Wnt signaling pathway, TF *Sox*, and *Ihh* (Figure 1).

It has been shown that the exosomes and extracellular vesicles of MSCs (MSC-Exos and MSC-EVs, respectively) have similar functions to MSC transplantation [49]. BMSC-EVs enhanced the expression of chondrogenic genes, type II collagen, and SOX9, and promoted the phenotypic transformation of synovial macrophages from M1 to M2 in cell cultures of RAW264.7 cells and chondrocytes [50]. MSC-Exos could prevent intervertebral disc degeneration by inhibiting apoptosis and promoting the proliferation of nucleus pulposus cells, inhibiting ECM degradation, alleviating the inflammatory response and oxidative stress, promoting chondrogenic differentiation, and protecting endplate chondrocytes and annulus fibrosus [51]. Moreover, MSC-Exos and MSC-EVs are promising as deliverers of targeted therapy [52]. At present, the commonly used targeted modification approach is to use genetic engineering to transfect the gene encoding the targeted peptide into exosome source cells, resulting in exosomes carrying the targeted peptide [53]. Other approaches may include the insertion into MSC-EVs of cell-penetrating peptides (CPPs), (viral) fusogenic peptides/proteins, and cationic lipids. They are currently under investigation [54].

## 5. Peptides That Stimulate Chondrogenic Differentiation

A variety of different molecular factors involved in the chondrogenic differentiation of MSCs open up wide possibilities for its regulation. In existing protocols for chondrogenic differentiation, growth factors, mainly TGF-β, BMP, IGF, and FGF, are usually added to the culture medium. The influence of growth factors on chondrogenesis may vary depending on the dose, cell type, and differentiation stage. However, stimulation of MSCs with growth factors usually requires their high concentration and repeated administration, which can result in side effects [55].

A safer and more physiological way to stimulate MSC differentiation into chondrocytes is to use peptides. One of the common approaches to imitate the physiological environment is the functionalization of biomaterial surfaces using peptides derived from the ECM, which are capable of recruiting stem cells and triggering their differentiation. Peptides of various lengths are used as scaffold biomaterials and for triggering various signaling pathways in the tissue engineering of the cartilage [56].

B2A is a synthetic peptide that modulates the activity of the BMP-2 factor. Its amino acid sequence was derived from the structure of BMP-2 and includes three domains: the heparin-binding, hydrophobic, and receptor target domains [57]. B2A peptides bind to type I and type II BMP receptors and induce chondrocyte differentiation and cartilage repair. In mouse stem cell cultures, the addition of the B2A peptide induced the expression of 11 genes associated with the following signaling pathways of chondrogenic differentiation: *Fgf*, *Fgfr1*, *Fgfr2*, *Smad1*, *Smad4*, *Twist1*, *Col11a1*, *Col3a1*, *Phex*, *Serphin1*, and *Bmp1*. The addition of the B2A peptide activated the proliferation of chondrocytes in vitro and led to an increase in the ECM components (sulfated glycosaminoglycan and collagen). In a knee joint OA model in rats, the administration of B2A into the synovial space enhanced cartilage repair compared with the control without B2A administration [58].

The casein kinase II (CK2) protein interacts with the type Ia BMP receptor (BMPRIa). The binding of BMP2 to BMPRIa releases CK2 and activates the smad1/5/8 pathway. Three peptides, CK2.1, CK2.2, and CK2.3, were synthesized that inhibit CK2 binding to BMPRIa and activate the BMP signaling pathway in the absence of a BMP ligand. It has been shown that CK2.2 and CK2.3 trigger different pathways of C2C12 cell line differentiation: the addition of CK2.3 led to osteogenesis, while CK2.2 led to adipogenesis and osteogenesis. Apart from that, CK2.3 and CK2.2 activated the Smad signaling pathway. Initially, these peptides were studied as factors of osteogenesis regulation. It has now been established that they can activate non-canonical BMP signaling pathways. Thus, these peptides can be inducers of chondrogenic differentiation. The addition of the CK2.1 peptide to C3H10T1/2 cells led to an increase in the synthesis of proteoglycans and expression of type II collagen. Injections of the CK2.1 peptide stimulated the formation of articular cartilage in mice. At the same time, unlike BMP2, CK2.1 did not induce the expression of collagen X, which indicated the possibility of cartilage tissue formation without its hypertrophy [59].

The SPPEPS (Ser-Pro-Pro-Glu-Pro-Ser) peptide was developed from aggrecan and TGF-b3. SPPEPS is a region of latency-associated peptide LAP (ligand for integrins) in the N-terminal region of TGF-b3. Integrins are known to mediate signaling interactions between cells and the ECM and play an important role in cartilage tissue regeneration. The addition of the SPPEPS peptide to the BM-MSCs of rats resulted in an increase in the expression of the type II collagen gene and the Sox9 transcription factor. SPPEPS can also activate other genes associated with chondrogenesis, including ENPP1 and CLIC4 [60].

KLD-12 is a self-assembling peptide (SAP) of 12 amino acid residues (AcN-Lys-Leu-Asp-Leu-Lys-Leu-Asp-Leu-Lys-Leu-Asp-Leu-CNH(2)). The use of the KLD-12 peptide for the encapsulation of chondrocytes into 3D cultures has been demonstrated, in which the encapsulated chondrocytes retained the phenotype and produced the ECM with a predominance of type II collagen. KLD has also been used to encapsulate and introduce MSCs into the intervertebral space. The ability of these self-assembling peptide scaffolds to induce the chondrogenic differentiation of BM-MSC bone marrow stromal cells has been demonstrated. The hydrogel formed with KLD-12 can fill bone and cartilage defects in situ to full thickness and promote cartilage repair [20,61].

The addition of the N-cadherin mimetic peptide to the KLD-12 peptide also led to the formation of a hydrogel, in which encapsulated human MSCs demonstrated increased expression of chondrogenic markers and deposition of a cartilage-specific ECM rich in proteoglycan and type II collagen. In the case of hydrogels formed with the participation of the N-cadherin mimetic peptide, chondrogenesis induction in human MSCs occurred upon suppression of the Wnt signaling pathway. This effect was achieved by reducing the translocation of β-catenin to the nucleus and inhibiting the activity of the β-catenin/LEF-1/TCF transcription complex [62].

One of the ways to cultivate MSCs is to add them to an alginate solution, which is a matrix for the formation of spheres during 3D cultivation. Cell adhesion peptides are able to improve the interaction of cells with matrix molecules. These peptides include RGD (Arg-Gly-Asp) and fibronectin mimetic peptide. In RGD-immobilized alginate scaffolds, the activation of both the Smad-dependent (SMAD2) and Smad-independent (ERK1/2) signaling pathways induced by TGF-β1 was more pronounced compared with cultures without RGD addition [63]. Alginates covalently bound to RGD have been shown to initiate interactions between alginate hydrogels and cells in culture [64].

A comparative study of the cultivation of human BM-MSCs with a collagen triple helix mimetic, GFOGER peptide (Gly-Pro-Cys-(Gly-Pro-Pro)5-Gly-Phe-Gly-Glu-Arg-(Gly-Pro-Pro)5-Gly-Pro-Cys-NH2), and RGD peptide was carried out in degradable gels. The GFOGER peptide increased proliferative activity and stimulated the synthesis of type II collagen in MSCs. The addition of GFOGER and RGD peptides increased the glycosaminoglycan production in MSCs [65].

The LPP peptide is a proteolytic N-terminal fragment of a link protein containing 16 amino acid residues (Asp-His-Leu-Ser-Asp-Asn-Tyr-Thr-Leu-Asp-His-Asp-Arg-Ala-Ile-His). LPP is a stabilizer of the main structural components of the cartilage: aggrecan and hyaluronic acid. LPP promotes ECM production in the intervertebral disc by upregulating SOX9, aggrecan, and type II collagen. This occurs through the interaction of the LPP peptide with the type II BMP receptor, which mediates signaling through the Smad pathway and results in the expression of BMP-4 and BMP-7. BMP-4 and BMP-7 enhance the Smad1/5 pathway signaling through the type I BMP receptor, promoting the expression of the SOX9 transcription factor alongside the aggrecan and type II collagen genes regulated by it. It is suggested that LPP may act as a therapeutic substitute for direct BMP administration in the treatment of intervertebral disc degeneration [66].

Collagen mimetic peptides (CMPs) have also been studied as stimulators of the chondrogenic differentiation of MSCs. In particular, the combination of CMPs with polyethylene oxide diacrylate (PEODA) formed a hybrid polymer–peptide framework, which, when added to the MSC cell culture, activated the synthesis of ECM components: glycosaminoglycans and collagen. MSCs cultured in a hydrogel supplemented with CMP/PEODA had a lower expression level of type X collagen, a marker of hypertrophy [67]. However, judging by the small number of publications on CMPs and their induction of MSC chondrogenesis, this method has not gained wide acceptance.

A polypeptide complex isolated from animal cartilage tissue (PCC) has been developed at the St. Petersburg Institute of Bioregulation and Gerontology. The PCC is a regulator of cartilage and bone tissue repair [68]. Currently, the PCC is being investigated as a chondroprotector in various models of pathology, including OA. This indicates the prospects for the study of the PCC as a substance that can potentially be effective in OA and have geroprotective properties. The PPC is currently in the second phase of clinical trials of its effectiveness during OA in Russia. The composition of the PCC includes short peptides with a molecular weight from 75 to 846 Da, including the tripeptide AED (Ala-Glu-Asp) [69,70]. The AED peptide stimulates the expression of the NFκB gene in replicative and stationary models of MSC aging. Since NFκB is a transcription factor that regulates inflammation, it can be suggested that the effect of the AED peptide on NFκB gene expression accelerates MSC aging and can reduce inflammation during OA. The AED peptide stimulates IFG1 gene expression in replicative and stationary MSC aging models. IGF1 is a growth hormone intermediator. It can be suggested that the AED peptide stimulates synthetic processes in aged MSCs. The AED peptide also affects TNKS2 gene expression in a replicative model of MSC aging. The TNKS2 gene product, tankyrase 2, is a member of the poly(ADP-ribose) polymerase family involved in supporting the telomere structure and regulating Wnt signaling pathway activity, as well as glucose metabolism, mitotic cycles, and many other processes. Higher expression of TNKS2 may be due to the multifunctional nature of tankyrases, which not only support the structure and integrity of telomeres but are also involved in regulating cell metabolism and growth. Thus, the AED peptide can regulate MSC metabolism and their differentiation [9]. It is known that chondrocytes are cells that have similarities in structure and function with fibroblasts. It was shown that the AED peptide regulates the synthesis of the Ki67 proliferation protein, the CD98hc glycoprotein, Caspase-3, and MMP9 in a model of the replicative aging of rat skin fibroblasts [7]. These data may also demonstrate the reparative properties of the AED peptide in cartilage tissue.

Thus, various types of short peptides have demonstrated regulating functions in the chondrogenic differentiation of MSCs. A summary of these functions is presented in Table 1.

## 6. Conclusions

Tissue bioengineering methods open up new prospects for therapeutic approaches to cartilage tissue regeneration in OA. Understanding the molecular mechanisms of chondrogenesis is crucial for the improvement of existing protocols for MSC chondrogenic differentiation. The search for biologically active substances capable of inducing the chondrogenic differentiation of MSCs is an urgent task for modern molecular medicine. The development of effective and safe peptide-based chondrocyte differentiation stimulators will contribute to the improvement of the quality of life in patients of older age groups with OA.

**Figure 2:**
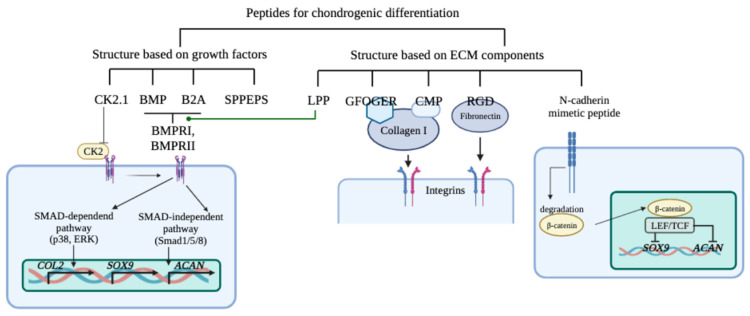
Molecular mechanisms of peptide regulation of MSC chondrogenic differentiation.

It has been established that synthetic peptide molecules of various lengths and functionalities mediate the induction of MSC chondrogenesis. Some of them are synthesized based on the TF structure and the others are based on ECM molecules (Figure 2). Thus, the chondrogenic differentiation of MSCs can be regulated by combining different approaches, depending on the targeted result.

The BMP and B2A peptides interact with BMP receptors. B2A triggers a predominantly SMAD-independent signaling pathway, while BMP enhances SMAD-dependent signaling. In addition, the CK2 phosphorylation site, the CK2.1 peptide, also activates BMP signaling. As a result, the expression of type II collagen, Sox9 transcription factor, and aggrecan increases, which leads to the chondrogenic differentiation of MSCs and increased chondrogenesis. The GFOGER and CMP peptides form a complex with type I collagen, while the RGD peptide forms a complex with fibronectin. These complexes interact with various integrin chains, enhancing the intercellular interactions necessary for the differentiation and proliferation of chondrocytes. The N-cadherin mimetic peptide causes the degradation of beta-catenin in the cytoplasm, resulting in a decrease in its translocation to the nucleus and a decrease in the transcription activity of the b-catenin/LEF-1/TCF complex, which inhibits the expression of Sox9 and aggrecan. The chondro- and geroprotective AED peptide can also regulate the activity of MSCs and their chondrogenic differentiation.

The presented data indicate prospects for further studies on biologically active peptides for the development of agents that stimulate the differentiation of chondrocytes and promote the repair of cartilage tissue in OA.

## Figures and Tables

**Table 1:** Peptides regulating chondrogenic differentiation of MSCs.

Peptide	Structure	Function
B2A	-	Binds to type I and type II BMP receptors [57]; Induces expression of *Fgf*, *Fgfr1*, *Fgfr2*, *Smad1*, *Smad4*, *Twist1*, *Col11a1*, *Col3a1*, *Phex*, *Serphin1*, and *Bmp1* [57]; Stimulates production of sulfated glycosaminoglycan and collagen [58]; Enhances cartilage repair [58].
CK2.1 CK2.2 CK2.3	-	CK2.2 and CK2.3 trigger C2C12 cell line differentiation [59]; CK2.3 stimulates osteogenesis [59]; CK2.2 stimulates adipogenesis and osteogenesis; CK2.3 and CK2.2 activate the Smad signaling pathway; CK2.1 stimulates the synthesis of proteoglycans and expression of type II collagen in C3H10T1/2 cells [59]; Injections of the CK2.1 peptide stimulate the formation of articular cartilage in mice [59].
SPPEPS	Ser-Pro-Pro-Glu-Pro-Ser	Stimulates the expression of the type II collagen gene and *Sox9* transcription factor [60]; Activates genes associated with chondrogenesis, including ENPP1 and CLIC4 [60].
KLD-12	AcN- Lys-Leu-Asp-Leu-Lys-Leu-Asp-Leu-Lys-Leu-Asp-Leu-CNH(2)	Stimulates the production of type II collagen by chondrocytes in cell culture; Induces chondrogenic differentiation of BM-MSC [20,61]; In combination with N-cadherin mimetic peptide, forms a hydrogel that increases expression of chondrogenic markers and deposition of cartilage-specific ECM rich in proteoglycan and type II collagen [62].
RGD	Arg-Gly-Asp	RGD-immobilized alginate scaffolds’ activation of SMAD2 and ERK1/2 pathways induced by TGF-β1 was more pronounced compared with cultures without RGD addition [63].
GFOGER	(Gly-Pro-Cys-(Gly-Pro-Pro)5-Gly-Phe-Gly-Glu-Arg-(Gly-Pro-Pro)5-Gly-Pro-Cys-NH2),	Increases proliferative activity and stimulates the synthesis of type II collagen in MSCs [65].
LPP	Asp-His-Leu-Ser-Asp-Asn-Tyr-Thr-Leu-Asp-His-Asp-Arg-Ala-Ile-His	Stabilizes aggrecan and hyaluronic acid; Promotes ECM production in the intervertebral disc by upregulating SOX9, aggrecan, and type II collagen; Interacts with type II BMP receptor that enhances Smad1/5 and promotes the expression of the SOX9 transcription factor alongside aggrecan and type II collagen genes regulated by it [66].
CMPs		Combination of CMPs with polyethylene oxide diacrylate (PEODA) activates the synthesis of ECM components: glycosaminoglycans and collagen in MSCs; MSCs cultured in a hydrogel supplemented with CMP/PEODA had a lower expression level of type X collagen, a marker of hypertrophy [67].
AED	Ala-Glu-Asp	Stimulates the expression NFκB gene in replicative and stationary models of MSC aging; Stimulates IFG1 gene expression during replicative and stationary MSC aging models; Affects TNKS2 gene expression in replicative model of MSC aging [9]; Regulates the synthesis of Ki67 proliferation protein, CD98hc glycoprotein, caspase-3, and MMP9 in a model of replicative aging of rat skin fibroblasts [7].

## Data Availability

The data is unavailable due to privacy.
